# The Use of the Voice Trainer App for Vocal Control in People with a Degenerative Ataxia: A Pilot Intervention Study

**DOI:** 10.1007/s12311-024-01662-5

**Published:** 2024-01-29

**Authors:** S. Knuijt, J. Nonnekes, B. P. C. van de Warrenburg, M. Nijkamp, S. Scholten, B. J. M. de Swart, J. G. Kalf

**Affiliations:** 1 Department of Rehabilitation, Donders Institute for Brain, Cognition and Behavior, Radboud University Medical Center, Nijmegen, the Netherlands; 2 Sint Maartenskliniek, Department of Rehabilitation, Nijmegen, the Netherlands; 3 Department of Neurology, Donders Institute for Brain, Cognition and Behavior, Radboud University Medical Center, Nijmegen, the Netherlands; 4 Voice and Swallowing Center Parkstad, Heerlen, the Netherlands

**Keywords:** Speech therapy, Ataxic dysarthria, Progressive ataxia, Vocal control, Voice trainer app

## Abstract

Dysarthria is disabling in persons with degenerative ataxia. There is limited evidence for speech therapy interventions. In this pilot study, we used the Voice trainer app, which was originally developed for patients with Parkinson’s disease, as a feedback tool for vocal control. We hypothesized that patients with ataxic dysarthria would benefit from the Voice trainer app to better control their loudness and pitch, resulting in a lower speaking rate and better intelligibility. This intervention study consisted of five therapy sessions of 30 min within 3 weeks using the principles of the Pitch Limiting Voice Treatment. Patients received real-time visual feedback on loudness and pitch during the exercises. Besides, they were encouraged to practice at home or to use the Voice trainer in daily life. We used observer-rated and patient-rated outcome measures. The primary outcome measure was intelligibility, as measured by the Dutch sentence intelligibility test. Twenty-one out of 25 included patients with degenerative ataxia completed the therapy. We found no statistically significant improvements in intelligibility (*p* = .56). However, after the intervention, patients were speaking slower (*p* = .03) and the pause durations were longer (*p* < .001). The patients were satisfied about using the app. At the group level, we found no evidence for an effect of the Voice trainer app on intelligibility in degenerative ataxia. Because of the heterogeneity of ataxic dysarthria, a more tailor-made rather than generic intervention seems warranted.

## Introduction

Ataxic dysarthria is common in people with ataxia and is characterized by slurred speech, imprecise articulation, and disturbed intonation patterns and rhythm [1]. A survey by Ataxia UK revealed that speech problems are one of the most frequent symptoms with a high impact [2]. Four recent studies demonstrated that a relatively short and intensive speech therapy program can accomplish positive changes in speech or psychosocial aspects in patients with degenerative ataxia [3–6]. However, results were inconsistent and the stability or duration of the reported improvements is unknown. A recent study in 21 ataxic patients reported that a short intensive treatment with LSVT LOUD improved the phonation duration and might have improved vocal quality, but intelligibility and naturalness evaluations showed no changes post-treatment [4]. In contrast, another study involving seven patients with ARSACS showed the opposite: the intelligibility of the patients improved after 4 weeks of intensive training, but there were no statistically significant changes in acoustic measurements of vocal quality [5]. When the same treatment was offered to patients with spinocerebellar ataxias due to CAG repeat expansions, both intelligibility and acoustic measurements improved [6]. In a mixed individual-group therapy, there were some improvements in intelligibility, but the most consistent effect was at the psychosocial level, with improvement of communication participation and confidence [3]. Based on these studies, speech therapy has the potential to accomplish meaningful changes in degenerative ataxias.

In the Netherlands, the Voice trainer app was originally designed to give patients with Parkinson’s disease (PD) real-time visual feedback on their loudness and pitch during a high-intensive program called the Pitch Limiting Voice Treatment (PLVT) [7]. Like in LSVT LOUD, patients are trained to overcome their hypokinetic speech by talking louder without raising their pitch. The Voice trainer app (produced by Elastique BV [8]) supports the patients during the treatment exercises and stimulates them to use their improved speech technique in daily life [9]. We hypothesized that patients with ataxic dysarthria would benefit from the Voice trainer app. More specifically, a better control of loudness and pitch may result in a lower speaking rate and thereby a better intelligibility.

The purpose of the current pilot study is to evaluate the use of the Voice trainer app and explore its effect on intelligibility in patients with degenerative ataxia.

## Materials and Methods

### Study Design

We used a pilot study with a pretest–posttest, single cohort design. Patients were assessed immediately prior to the training (T0), immediately after the training (T1), and 3 months after the training (T2). The assessments at T0 and T1 took place at the Radboudumc; the assessment at T2 was online using a reliable, certified, and secured online platform (software produced by Zaurus B.V. [10]) because of the long traveling distance for most patients.

### Patients

Patients with pure ataxic dysarthria due to degenerative ataxia were included in the outpatient clinic of the Department of Rehabilitation and the Radboudumc Expert Center for Rare and Genetic Movement Disorders between September 2020 and October 2021. They received the study information by email. Patients were called 1 week later to ask if they were willing to participate.

Inclusion criteria were the presence of a degenerative ataxia, age > 18 years, and absence of any other voice or speech disorder. All patients signed informed consent prior to the therapy.

Patient characteristics were collected from the medical files and included age, sex, and diagnosis. Because of the COVID-19 lockdown, patients had medical consultations by phone and therefore no up-to-date score on the Scale for Assessment and Rating of Ataxia [11]. Instead, we classified disease severity in accordance with previously published milestones: 0 = no gait disability, 1 = disease onset, as defined by the onset of gait difficulties, 2 = loss of independent gait, 3 = confinement to wheelchair, 4 = death [12].

None of the patients received speech therapy prior to or during the intervention and they did not receive other pharmaceutical therapies.

### Intervention

Treatment was delivered by four skilled speech-language therapists (SLTs). The therapy consisted of five therapy sessions of 30 min which had to be completed within 3 weeks. Based on our experience with the Voice trainer app in PD, we assumed that patients were able to apply the vocal control technique in five sessions, because of the real-time visual feedback. Due to COVID-19 and traveling distances, patients had the opportunity to choose for in-person or online treatment.

**Fig. 1:**
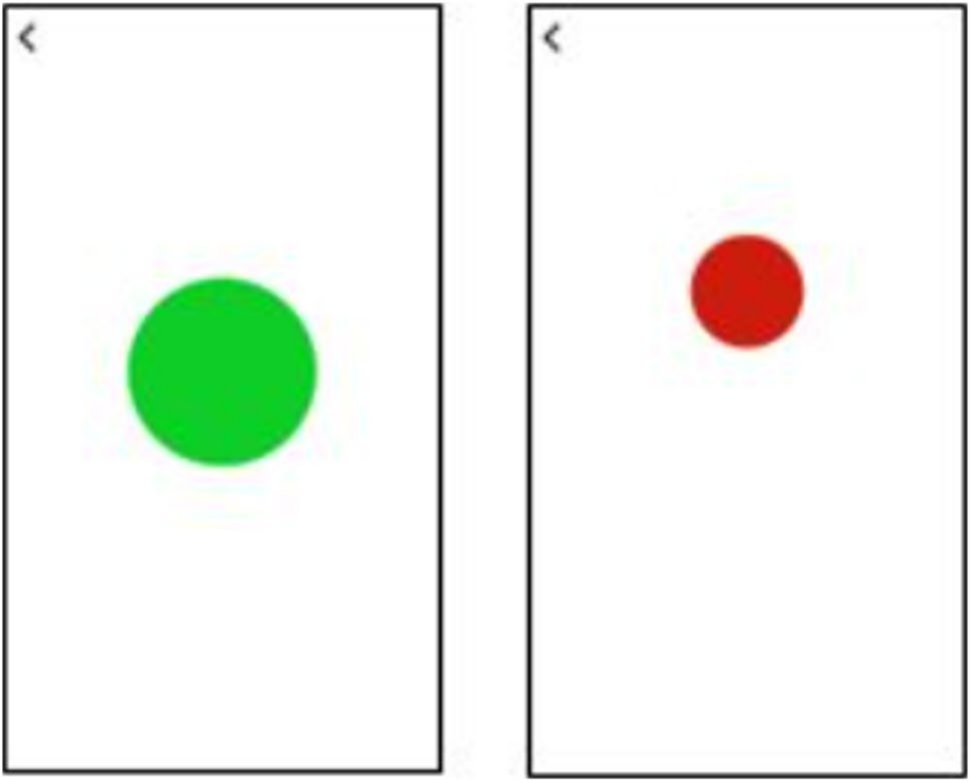
Two examples of screenshots of the Voice trainer app. Green and in the center = adequate loudness and pitch; red and above the center = too soft and too high

The Voice trainer app provides real-time visual feedback with a moving dot: the high-low movement of the dot reflects the pitch and the color and the size of the dot reflect the loudness—green is adequate loudness and red means that the voice is too soft (Fig. 1). The limits for loudness and pitch were personalized in the settings of the app by the SLT. In contrast to the therapy in PD, we did not stimulate the patients to use a loud voice, but to control the loudness at an acceptable level and to prevent excessive loudness and pitch variations.

The therapy is based on the principles of the Pitch Limiting Voice Treatment (PLVT) [7], the standard therapy for patients with PD in the Netherlands. At the start of the therapy, there was attention for optimal head and body position, and when required, patients did some stretching or relaxation exercises. The following exercise levels were subsequently practiced at an acceptable loudness and pitch:

- Level 1: the patient is able to produce an /a:/ and sequences (e.g., counting, days of the week).
- Level 2: the patient is able to produce short answers (e.g., yes, the sun is shining).
- Level 3: the patient is able to produce short spontaneous sentences.
- Level 4: the patient is able to produce answers on open questions.
- Level 5: the patient is able to have a conversation.

Patients were encouraged to practice at home at the level discussed or to use the Voice trainer in daily life, always in situations that were individually appropriate (e.g., telephone call, talking with a stranger). If possible, a co-trainer was instructed to support and instruct the patient.

### Observer-Rated Outcome Measures

#### Intelligibility

The primary outcome measure was intelligibility measured by the Dutch sentence intelligibility test (NSVO-Z) [13]. This test consists of reading aloud 18 semantically unpredictable sentences, randomly generated from a database of 1200 sentences. The sets included three sentences of each of the following lengths: 5, 7, 9, 11, 13, and 15 syllables. The test was scored by an independent SLT who was not blinded for the timepoint of the assessment. A total score of 100% intelligibility can be achieved, with a cut-off score of 96% for dysarthria, and a relative difference of > 5% represents a perceivable change [14].

#### Dysarthria Severity

The Radboud Dysarthria Assessment (RDA) [15] includes a perceptual rating of dysarthria severity based on six speaking tasks, i.e., spontaneous speech, reading, repetition rate, maximum phonation time, maximum phonation volume, and fundamental frequency range.

One SLT (SK) perceptually rated the dysarthria severity from the audiotape with 0 = no dysarthria, 1 = minimal dysarthria, 2 = mild dysarthria, 3 = moderate dysarthria, 4 = severe dysarthria, and 5 = very severe dysarthria/anarthria. She checked this score with the SLT who treated the specific patient.

#### Acoustic Measurements

Acoustic measurements were derived from the standardized reading text of the RDA. Using the online speech analysis software program PRAAT [16], we measured articulation and speaking rate, pause duration, amount of pauses more than 300 ms, habitual (median) speaking fundamental frequency, and also vocal quality expressed in the harmonic-to-noise ratio (HNR) measured on a 3-s mid-segment of an /a:/ phonation. Because of the reduced quality of the online recordings at T2, we had to exclude the vocal quality measurement in T2.

#### Naturalness of Speech

The naturalness of speech was assessed using two rater-blinded listening experiments.

In the first experiment, 12 raters perceptually rated a standard sentence from the text on naturalness using direct magnitude estimation (DME) [17]. In DME, a reference stimulus of a mild dysarthria is presented to the listeners, given a score of 100. The raters score the naturalness of speech of the treated patients in comparison to the reference stimulus. A score > 100 is assigned to patients who sound more natural than the reference stimulus and a score < 100 to patients who sound less natural. Nine blocks of four sentences (after every block, the reference stimulus was presented) were randomly presented to the listeners. Six sentences were presented twice for reliability measurements.

The second experiment was a paired-comparison (PC) task. Thirteen raters heard two standard sentences of the text and were asked which sentence sounded more natural (the first, the second, or no preference). Twenty pairs were presented. There were two versions of this task in which the order of T0 and T1 was mirrored in case the raters had a preference to score the first or the last sentence.

### Patient- and Caregiver-Rated Outcome Measures

#### Dysarthria Questionnaire

The self-evaluation questionnaire of the RDA includes seven questions about functions, activities, and participation, yielding a total score from 7 (no complaints) to 35 (most severe complaints).

#### Satisfaction

Satisfaction about speech quality was scored by the patient and a family member or close friend, on a visual analogue scale from 0 to 10.

#### Communicative Participation

Patients filled in the Dutch Communicative Participation Item Bank–short form (CPIB-10) [18], a validated questionnaire consisting of ten questions about communicative participation. The total score ranges from 10 (no problems) to 40 (most severe problems).

#### Feasibility

At T1 and T2, participants completed a short questionnaire about the use of the Voice trainer. The questionnaire consisted of open-ended and closed-ended questions about the use of the Voice trainer to apply the speaking technique (in which situations are you using the Voice trainer), the importance of using the Voice trainer to learn the new speaking technique (VAS), and satisfaction with the intensity of the treatment (too short, long enough, too long).

### Statistical Analysis

First, descriptive statistics were performed on the different outcome measures. We then performed a repeated-measures ANOVA, with *time* as within-subjects factors (T0, T1, and T2). In case of a significant effect, post hoc testing using paired samples t-tests was performed. Intraclass correlation coefficients (ICC) were used for interrater reliability measurements in the DME listening experiment. In the paired-comparison listening experiment, a chi-square test was used to measure if there was a consistency in scoring the preferred sentence (T0, T1).

SPSS statistics 25 for Windows (IBM Corp., Armonk, NY) was used for statistical analysis and we accepted a *p*-value of < 0.05 as statistically significant.

## Results

### Patients

In total, 25 patients were included. Four patients dropped out because of different reasons (1 lost in follow-up, 2 were too busy to continue, 1 preferred speech therapy in primary care), leaving 21 patients for analysis.

We included 11 men (52%) and 10 women (48%) with a mean age of 56 years (range 21–80). The mean age of the men was 71 years (range 52–80) and of the women 41 years (range 21–57). For patient characteristics, see Table 1.

**Table 1:** Patient characteristics

Patient	Sex	Age	Diagnosis	Genetics	Disease stage*
2	F	53	SCA36	NOP56	1
3	F	57	ILOCA	n.a	2
4	F	45	ILOCA	n.a	1
5	M	75	ILOCA	MSK1	3
6	F	27	Friedreich ataxia	n.a	3
7	F	55	SCA6	22 repeats	2
8	F	29	Friedreich ataxia	n.a	3
9	M	72	ILOCA	CD99L2	1
10	F	38	Friedreich ataxia	n.a	1
12	F	26	ADCA	SYNE1	1
15	M	73	SCA14	n.a	2
16	M	63	ILOCA	ERCC4	1
17	M	70	SCA3	n.a	2
18	M	63	ILOCA	n.a	1
19	F	21	Friedreich ataxia	n.a	1
20	M	78	SCA23	PDYN	2
21	M	80	SCA23	PDYN	2
22	M	79	SCA14	PKCγ	2
23	M	52	SCA2	39 repeats	1
25	F	56	ADCA	n.a	3
26	M	73	ILOCA	RFC1	2

*SCA*, spinocerebellar ataxia; *ILOCA*, idiopathic late-onset cerebellar ataxia; *ADCA*, autosomal dominant cerebellar ataxia (no known genotype); *n.a.*, not available

*1 = onset of gait difficulties, 2 = loss of independent gait, 3 = confinement to wheelchair

### Observer-Rated Outcome Measures

#### Intelligibility

Intelligibility scores did not change significantly over time (time, *F*(2,38) = 0.59, *p* = 0.56) (Table 2).

**Table 2:** Overview of all outcome measures

Outcome measure	T0 (prior to the treatment), mean (range)	T1 (immediately after the treatment), mean (range)	T2 (three months after the treatment), mean (range)
Intelligibility (%)	91.1 (71.8–100)	92.2 (72.2–100)	92.7 (72.8–100)
Articulation rate (syll/s)	3.5 (2.3–4.1)	3.4 (2.4–4.4)	3.3 (2.4–4.4)
Speaking rate (syll/s)	2.3 (1.7–3.2)	2.2 (1.6–3.0)*	2.2 (1.3–2.8)
Pauses (n)	102.4 (17–187)	112.3 (15–250)	117.5 (14–187)
Mean pause duration (ms)	1346.5 (810–2350)	1530.0 (960–2540)*	1300.0 (700–2220)
Fundamental frequency (Hz)			
Men	136.5 (111.1–177.3)	133.6 (112.5–191.7)	-
Women	206.7 (161.1–237.8)	195.4 (136.1–268.5)	
Harmonic-to-noise ratio (dB)	16.03 (4.5–24.5)	14.66 (6.5–23.1)	-
Naturalness (DME)	81.2 (25–151)	81.1 (21–145)	-
Questionnaire RDA (7–35)	13.15 (7–28)	12.0 (6–28)	10.8 (7–17)
Communicative participation (10–40)	27.3 (19–38)	25.9 (16–40)	25.1 (16–33)
Satisfaction patient (VAS 0–10)	4.5 (0–7)	4.9 (0–9.6)	4.6 (0–8)
Satisfaction family member (VAS 0–10)	5.3 (0–9)	5.2 (0–9.6)	5.6 (0.2–8.5)

*RDA*, Radboud Dysarthria Assessment; *DME*, direct magnitude estimation; *syll/s*, syllables per second; *statistically significant effect between T0 and T1

Fifteen patients (71%) scored below the cut-off score of 96% on the NSVO-Z. Their mean score improved from 88.6% on T0, to 89.5% on T1, to 91.7% on T2. Post hoc testing revealed that the difference between T0 and T2 is statistically significant (− 3.01 (95% CI − 5.95 to − 0.07), *p* = 0.045).

Of the nine patients with a positive change, four scored a perceivable improvement of > 5% between T0 and T1, which remained at T2.

#### Dysarthria Severity

At T0, seven patients were scored as mild dysarthria, nine moderate, and five severe. At T1, no changes in dysarthria severity were observed compared to T0.

#### Acoustic Measurements

The mean speaking rate was significantly slower over time (*time*, *F*(2,30) = 3.83, *p* = 0.03) (Table 2). Post hoc testing revealed that the difference between T0 and T1 was statistically significant (0.14 (95% CI 0.03 to 0.25), *p* = 0.02). The mean pause duration was longer (*time*, *F*(2,30) = 11, *p* < 0.001). Post hoc testing revealed that the difference between T0 and T1 was statistically significant (− 30.57 (95% CI − 60.58 to − 0.56), *p* = 0.05).

#### Naturalness

Seventeen patients were included in the experiments, because the quality of the recordings had to be sufficient for this goal. We only compared T0 with T1, because the recordings of T2 were recorded via Zaurus, leading to a reduced quality. The interrater reliability of the DME resulted in an ICC of 0.92 (95% CI = 0.87–0.96) (*p* < 0.001). There was no statistically significant difference in the mean DME score between T0 and T1 (*z* =  − 0.28, *p* = 0.977) (Table 2). In the paired-comparison experiment, raters had in 43.8% of the pairs a preference for T0 and in 31.7% for T1, and in 24.5%, there was no preference. The chi-square test revealed no significant preference for T1 over T0 (*χ*^*2*^ (2) = 0.34, *p* = 0.46).

### Patient- and Caregiver-Rated Outcome Measures

#### Dysarthria Questionnaire

There were no statistically significant changes between the three assessments (*time*, *F*(2,36) = 1.90, *p* = 0.16) (Table 2).

#### Communicative Participation

Scores on communicative participation marginally improved, but the differences were statistically not significant (*time*, *F*(2,36) = 2.12, *p* = 0.13) (Table 2).

#### Satisfaction

Patient satisfaction about speech quality did not change over time (*time*, *F*(2,36) = 0.15, *p* = 0.86), nor did the satisfaction of the family member about the speech quality of the patient (*time*, *F*(2,36) = 0.46, *p* = 0.64) (Table 2).

#### Feasibility

Patients valued the use of the Voice trainer with a mean VAS score of 6.7 (median 7, range 2–10) on the question of how important the Voice trainer was in learning another speaking technique and on T2 6.4 (median 7.5; range 0–10).

Two patients stated that the Voice trainer did not help at all, but at follow-up (T2), 16 of the 19 patients (84%) still used the app in different situations, or to “calibrate” their speech.

Regarding treatment duration, 13 patients felt that the five sessions were enough, but eight patients needed more therapy sessions and were referred to a speech therapist in their region.

## Discussion

Our short and intensive therapy program to train patients with progressive ataxic dysarthria to use the Voice trainer app generated no statistically significant or clinically relevant improvements in intelligibility and communicative participation. However, patients tended to speak slower after the intervention, used more pauses, and in general were satisfied using the Voice trainer app during the therapy.

One explanation of the lack of improvement of intelligibility at the group level could be the individual differences between patients at baseline as well as after treatment: we included patients with different types of ataxia, their disease stage differed from onset of gait difficulties to confinement to wheelchair, their severity of dysarthria ranged from mild to severe, and they showed a wide range in intelligibility scores. This heterogenicity caused that we were not able to perform subgroup analysis to explain why in some patients the intelligibility improved, while in other patients, the intelligibility declined (and on T2 improved again). Another explanation might be relatively the high mean intelligibility score pre-treatment, generating limited room for improvement. Still, there was a significant improvement of intelligibility between T0 and T2 in patients with a score below 96% intelligibility, but only in four patients, this improvement was clinically relevant. Although we included patients with different levels of dysarthria severity, patients’ self-reported speech problems and social consequences were low. This discrepancy between clinician-rated dysarthria severity and intelligibility or patient-rated complaints is also known from previous research [19], which confirms that multiple domains should be measured at all times. Another possible explanation for the absence of general improvement is that most patients report fluctuations of their speech, e.g., during the day. Such intrinsic variability may prevent the detection of small statistically significant effects.

The naturalness of speech did also not improve in our population. The aim of using the Voice trainer app was to control the loudness and pitch, and in some patients, this might have resulted in more monotonous speech. One of them even reported that despite better intelligibility, she was afraid to lose the spontaneity in her voice. We used the reading text in the naturalness experiments to ensure that we could use the same sentences comparing T0 with T1. Hilger et al. found in a group of 27 patients with ataxic dysarthria that naturalness was higher in spontaneous speech compared to reading [19]. This suggests that we could have found different outcomes when we would have used samples of spontaneous speech in our analyses as well.

An often-used compensation by people with ataxic dysarthria is slowing down the speech rate for better articulation by using the so-called scanned speech (like “Cof-fee-with-milk-and-su-gar-please”). However, in scanned speech, the app has difficulties in registering the speech resulting in the disappearance of the dot. Stimulating the patients to keep the dot visible by for example lengthening the vowels improved the fluency. This was an effect we did not expect beforehand. However, in severe ataxic dysarthria, patients probably need to use scanned speech to be intelligible.

Regarding the length of treatment, most patients were content with the amount of therapy sessions. There were eight patients who required additional speech therapy sessions. They were referred to a speech therapist in their neighborhood. Another relevant finding was that the patients who still used the app on T2 did not use it as a visual feedback tool during social interaction, but to “calibrate” their speech and quickly regain control.

### Limitations

This pilot study has several limitations showing us valuable lessons. First, intelligibility was scored by an independent but experienced SLT who was unblinded for timepoint of the assessment. Blinding the assessor, using multiple raters, using inexperienced listeners, or using multitalker babble noise over speech could have given more insight in the complex process of speech perception [20, 21]. Second, we measured the patients on T1 and T2 without using the Voice trainer app, because we expected that they would be able to use the new speaking technique. However, to automate speech will take much longer than five sessions in 3 weeks [22], so it would have been more correct to use the app during the measurements. Third, the measurements were only taken once. When there may be instability in the speech, like in ataxic dysarthria, one can argue about the reproducibility of the measurements [23]. Fourth, the assessment at T2 was conducted online compared to T0 and T1. Although the quality of the recording was sufficient, we cannot rule out any effect of the different assessment procedures on the analysis. And finally, we did not register or control the amount of exercises at home. This could have made the therapy more intensive, having positive effects on the results. Besides, patients were free to choose in which way they used the app between T1 and T2, which makes it difficult to draw conclusions about the retention.

The Voice trainer app has been developed for patients with a hypokinetic dysarthria due to Parkinson’s disease (PD). In PD, a loud and low voice will immediately improve the quality of the speech [7]. Aspects of ataxic dysarthria are more diverse, maybe more complex, and therefore, more difficult to influence. The complexity of ataxic dysarthria might be the cause of the inconsistent findings between recent studies and the effect of the therapy programs on the individual patients. The speech characteristics in ataxic dysarthria differ between patients along the aspects of speech production (articulation, voice, nasal resonance, prosody, and breathing). Still, recent studies show that there can be positive effects of speech therapy interventions [3–6], but more than for any other dysarthria, we may have to apply individually tailored rather than standardized speech interventions for patients with ataxic dysarthria.

## Data Availability

The data that support the findings of this study are available on request from the corresponding author, SK. The data are not publicly available due to the privacy of the patients.
